# Mortality is not increased with Diabetes in hospitalised very old adults: a multi-site review

**DOI:** 10.1186/s12877-020-01913-0

**Published:** 2020-12-03

**Authors:** Peter Smerdely

**Affiliations:** 1 Department of Aged Care, St George Hospital, 3 Chapel Street, Kogarah, Sydney, NSW 2217 Australia; 2 School of Population Health, University of NSW, Sydney, Australia

**Keywords:** Health outcomes, Older adults, Hospital, Diabetes

## Abstract

**Background:**

Few data exist regarding hospital outcomes in people with diabetes aged beyond 75 years. This study aimed to explore the association of diabetes with hospital outcome in the very old patient.

**Methods:**

A retrospective review was conducted of all presentations of patients aged 65 years or more admitted to three Sydney teaching hospitals over 6 years (2012–2018), exploring primarily the outcomes of in-hospital mortality, and secondarily the outcomes of length of stay, the development of hospital-acquired adverse events and unplanned re-admission to hospital within 28 days of discharge. Demographic and outcome data, the presence of diabetes and comorbidities were determined from ICD10 coding within the hospital’s electronic medical record. Logistic and negative binomial regression models were used to assess the association of diabetes with outcome.

**Results:**

A total of 139,130 separations (mean age 80 years, range 65 to 107 years; 51% female) were included, with 49% having documented comorbidities and 26.1% a diagnosis of diabetes.

When compared to people without diabetes, diabetes was not associated with increased odds of mortality (OR: 0.89 SE (0.02), *p* < 0.001). Further, because of a significant interaction with age, diabetes was associated with decreased odds of mortality beyond 80 years of age. While people with diabetes overall had longer lengths of stay (10.2 days SD (13.4) v 9.4 days SD (12.3), *p* < 0.001), increasing age was associated with shorter lengths of stay in people aged more than 90 years. Diabetes was associated with increased odds of hospital-acquired adverse events (OR: 1.09 SE (0.02), *p* < 0.001) and but not 28-day re-admission (OR: 0.88 SE (0.18), *p* = 0.523).

**Conclusion:**

Diabetes has not been shown to have a negative impact on mortality or length of stay in hospitalised very old adults from data derived from hospital administrative records. This may allow a more measured application of diabetic guidelines in the very old hospitalised patient.

## Background

Diabetes mellitus is common in developed western nations, with 30 million people diagnosed with diabetes (prevalence 9.4%) in the US in 2015 [1], and prevalence of 6 and 5.4% in the UK and Australia, respectively [2].

Diabetes is associated with an increased risk of morbidity and mortality. Diabetes UK reports increased cardiovascular, renal, ophthalmic, peripheral vascular, neurological and psychiatric disease in poorly controlled diabetes with increased risk of mortality and reduced life expectancy [3]. In Australia, the Australian Institute of Health and Welfare (AIHW) reports death rates being between 1.6 and 2 times higher for those with diabetes than the general population [4].

Hospitalisation in people with diabetes is more likely, more frequent, and with a longer length of stay [5–7]. Consistent with this, in Australia, the prevalence of hospitalisation for any reason of people with diabetes ranges from between 8.9 to 35.1% [8, 9].

Ageing is strongly associated with the development of diabetes [10]. Likely, the interplay between genetics, environmental factors, and normal ageing is the cause [11]. The prevalence of diabetes increases with age, with 3–5 times the prevalence of diabetes in people over aged 65 years [1, 2].

However, detailed data about hospitalisation or outcomes in people with diabetes aged beyond 75 years are presently lacking, with results often grouped as 65+ or 75+ [12]. Anecdotally, the impression of experienced geriatricians is that diabetes in itself does not confer increased morbidity or mortality in very old adults. This impression occasionally causes conflict with other clinicians or family members when strict diabetic control is relaxed.

This study was undertaken to explore the effect of diabetes diagnosis and age on hospital mortality of patients aged over 65 years using hospital administrative data. The age of 65 years is commonly used to define the older adult. In addition, the hospital outcomes of length of stay (LOS), the development of hospital-acquired adverse events, and unplanned re-admission to hospital within 28 days of discharge was examined.

## Methods

### Study design

A retrospective study was conducted of patients aged 65 years and older admitted for 24 h or more for acute care in the South East Sydney Local Health District (SESLHD) over 76 months, from 1 July 2012 to 30 September 2018. The study excluded patients presenting for day-only intervention, outpatient reviews, routine renal dialysis, ambulatory care, and psychiatric management. Subjects were flagged if they had an admission in the 7 years prior to the study period. It was anticipated that patients might have multiple admissions over the study period.

### Setting

SESLHD is located in south east Sydney, Australia, comprising three acute care hospitals: Prince of Wales, St George, and Sutherland. These hospitals provide approximately 1420 beds for south east Sydney, with approximately 139,321 people over 65 years living in the district in 2016. The prevalence of diabetes in the district is 6.4% and is below the state (NSW) average prevalence of 8.7% [12].

### Data

Data for this study were based on hospital administrative data, derived from information obtained from the medical record of the patients. These administrative data contain demographic details such as age, sex and race, as well as diagnosis codes, derived from clinician entries in the medical record. This information is held within the “Patient Information Manager” (iPM). iPM is a patient administration system handling all the demographic data, discharge diagnostic codes (DRG), and separation data for all admissions. Study eligibility was based on coding within iPM. Cases were defined as those with a principal or additional diagnosis of diabetes (ICD-10-AM code: E10 Type 1 diabetes mellitus, E11 Type 2 diabetes mellitus, E12 Malnutrition-related diabetes mellitus, E13 Other specified diabetes mellitus, E14 Unspecified diabetes mellitus) as defined by International Classification of Disease 10th Revision Australian Modification (ICD-10-AM). Cases included a new diagnosis during the admission. For comparison, data was also collected from a non-diabetic cohort for the same period, for comparison. A modified Charlson Comorbidity Index (CCI) was generated from ICD-10-AM codes [13, 14]. The CCI was modified by removing the weights associated with diabetes.

The datasets used and/or analysed during the current study are available from the corresponding author on reasonable request.

### Hospital-acquired diagnosis (HADx)

The Classification of Hospital Acquired Diagnoses is a validated system that permits the identification of adverse events that have occurred in hospital during an admission using hospital administrative data [15, 16]. A hospital acquired diagnosis (HADx) index was generated using a custom written Stata program adapted by the author from existing programs [14]. HADx has been included as a possible confounder and outcome in this study.

### Unplanned re-admission

Unplanned re-admission was defined as being admitted within 28 days following a previous separation. Pre-arranged or booked admissions within this time frame were excluded.

### Statistical analysis

Data were analysed using Stata Version 16 (StataCorp, College Station, TX, USA). Variables are described using mean and standard deviation, proportion and range. Each separation was analysed as a unique observation. Age, presence of diabetes and the interaction of these two were the variables of interest. The outcome variables of interest were, primarily, mortality during hospital admission (now referred to as mortality), and secondarily, LOS, hospital-acquired disease and re-admission rates. T-tests, Chi-Square tests and Mann-Whitney tests were used where appropriate. Logistic and negative binomial regression models were used to assess association for the binary (mortality, 28-day re-admission and HADX) and count (LOS) outcomes. All models were adjusted for the potential confounding effects of sex, CCI, HADX, and number of admissions before the study period. Effect modification (interaction) was assessed between diabetes and the other variables in the model. A 5% two-sided significance level was used for main effects and 1% for interactions.

## Results

### Cohort summary

There were 191,201 hospital separations recorded between October 2012 and October 2018 in the district. Of these, 145,090 had a LOS greater than 24 h, from which 139,130 separations were further examined following exclusion of separations for routine renal dialysis and psychiatric management.

Table 1 summarises characteristics of this cohort. A total of 26.1% of the cohort was recorded as having diabetes. Half of the cohort (69,513) had at least one separation recorded in the 7 years before the study period. People with diabetes had a higher mean number of separations (4.2 (SD 3.9) v 3.5 (SD 3.6), *p* < 0.001). Ages ranged from 65 to 107 years.

**Table 1:** Cohort Characteristics

	Diabetes		No Diabetes		***p***^**α**^
Age					
Mean (Years, (SD))	78.8	(7.4)	80.4	(8.2)	< 0.001^β^
Median (Years, (IQR))	79	(73–84)	81	(74–87)	< 0.001^g^
Age Category (n, (%))^¥^					
65–69	4698	(27.7)	12,251	(72.3)	
70–74	6696	(29.2)	16,253	(70.8)	
75–79	8081	(30.6)	18,363	(69.4)	
80–84	8034	(28.1)	20,518	(71.9)	
85–89	5976	(23.0)	20,037	(77.0)	
90–95	2300	(16.3)	11,810	(83.7)	
95+	524	(12.7)	3589	(87.3)	
Female (n, (%))	16,026	(44.1)	55,030	(53.5)	< 0.001
Separations (n, (%)) ^£¥^	36,309	(26.1)	102,821	(73.9)	
1 Separation	16,208	(44.6)	53,192	(51.7)	< 0.001
2 Separations	7609	(21.0)	21,729	(21.1)	
3 or more Separations	12,492	(34.4)	29,900	(27.1)	
No Prior Separations^¥^	19,774	(54.5)	49,739	(48.4)	< 0.001
First Separation & No Prior^¥^	9323	(22.2)	32,697	(77.8)	
No. of Prior Separations ($\overline{\mathrm{x}}$(SD))	3.8	(3.7)	3.1	(3.4)	< 0.001^β^
CCI ^€^ (n, (%))	3361	(49.1)	11,427	(57.6)	< 0.001
	2481	(36.3)	6450	(32.5)	
	1001	(14.6)	1953	(9.9)	
Hospital Acquired Diagnosis©(n, (%))	9507	(26.2)	26,354	(23.5)	< 0.001
Adverse drug events	1431	(3.9)	3781	(3.7)	
Cardiovascular	1210	(3.2)	3209	(3.3)	
Intra and post procedural	1030	(2.8)	2899	(2.8)	
Other	842	(2.3)	2234	(2.2)	
Respiratory	840	(2.3)	1967	(1.9)	

Percentages are column percentages unless indicated. ¥Percentages are row percentages. £Number of separations by each individual in the 6-year period. €Charlson Comorbidity Index. α*P* values are from χ2, βP values are from t-test. **g** *P* values are from rank-sum test

There were 848 separate ICD10-AM codes in the cohort, with the five most frequent being: Acute myocardial infarction (code I21); Heart failure (I50); Cerebral infarction (I63); Pneumonia, unspecified organism (J18); and Fracture of femur (S72). These represent 64% of all diagnoses. CCI scores ranged from 0 to 12 (mean of 1.03 (SD 1.63)), with 55% scoring 0, 20% scoring 1, and a 29% scoring 2 or more on this measure.

People with diabetes were more likely to have a HADx (complication), see Table 1.

### Primary outcome

**Fig. 1:**
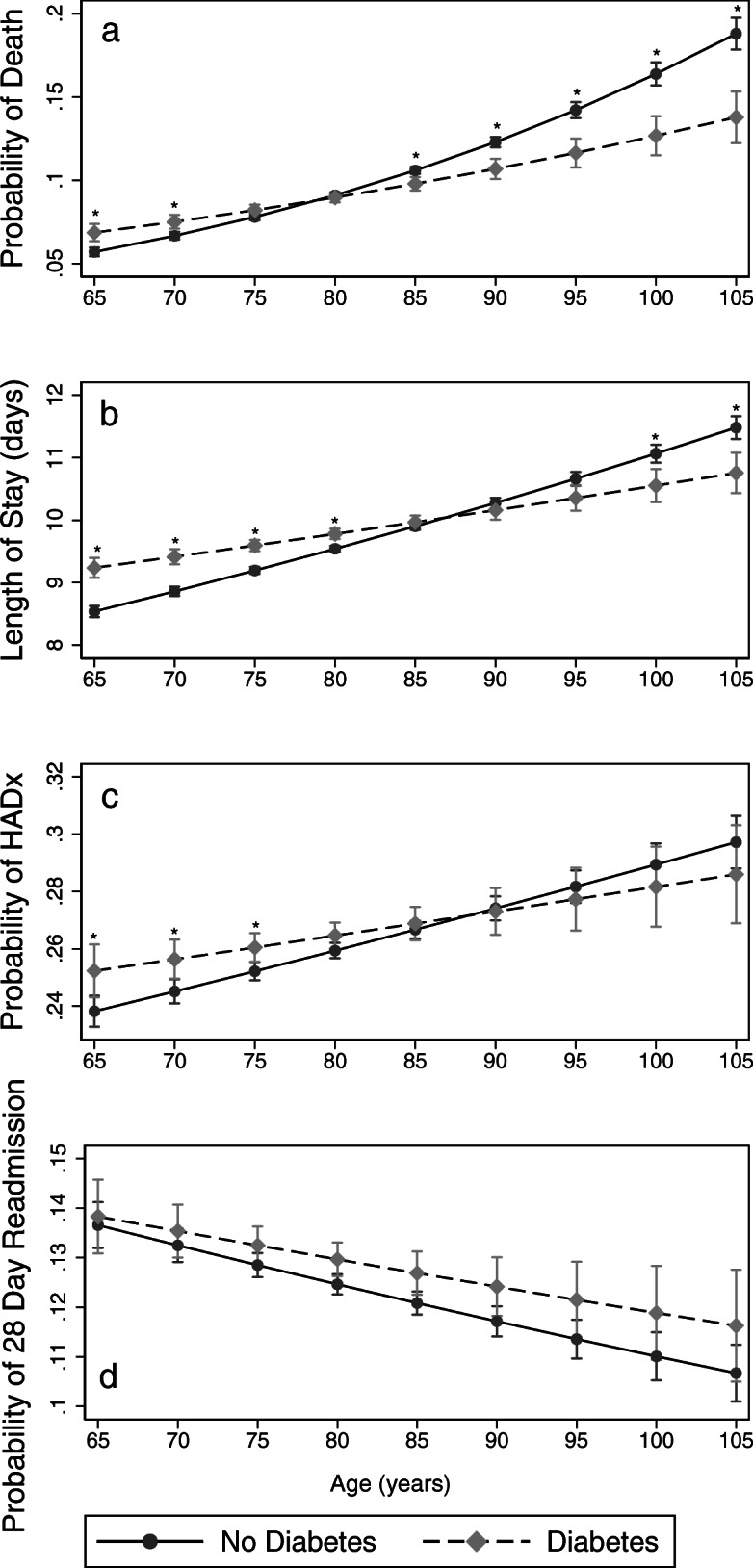
Predictive Margins of Diabetes by Age against various outcomes. **a**: Predictive Margins of Diabetes by Age for Death. **b**: Predictive Margins of Diabetes by Age for Length of Stay. **c**: Predictive Margins of Diabetes by Age for Hospital Acquired Diagnosis. **d**: Predictive Margins of Diabetes by Age for 28-day Readmission. Asterisk indicates *p* < 0.001 at the age

The mortality rate for people with diabetes was 8.6% (v 9.6% in non-diabetics) (crude odds ratio 0.89, *p* < 0.001), see Table 2. When adjusted for age and its interaction with age, the odds ratio was 2.98, and when further confounders were added to the analysis, the odds ratio was 3.15. The analysis demonstrates a significant interaction between age and diabetes. When using the predictive margins generated by the model, mortality in people with diabetes is lower after age 80 years, see Fig. 1a.

**Table 2:** Logistic Regression for Mortality

	Unadjusted			Model 1			Model 2		
	OR	SE	*p*	OR	SE	*p*	OR	se	*p*
Diabetes	0.89	0.02	< 0.001	2.98	0.68	< 0.001	3.15	0.72	< 0.001
Age	1.03	0.00	< 0.001	1.03	0.00	< 0.001	1.04	0.00	< 0.001
Diabetes x age				0.99	0.00	< 0.001	0.99	0.00	< 0.001
Female	1.26	0.02	< 0.001				1.18	0.02	< 0.001
Prior Separations(n)	0.94	0.00	< 0.001				0.94	0.00	< 0.001
Admission Number	0.92	0.00	< 0.001				0.93	0.01	< 0.001
CCI 1	0.72	0.02	< 0.001				0.71	0.02	< 0.001
CCI 2+	0.86	0.02	< 0.001				0.91	0.02	< 0.001
HADx	1.39	0.04	< 0.001				1.45	0.04	< 0.001
Constant				0.01	0.00	< 0.001	0.01	0.00	< 0.001

Abbreviations: *OR* Odds ratio; *se* Standard error; *CCI* Charlson Comorbidity Index; *HADx* Hospital acquired diagnosis. Models comprise components shown. Unadjusted model used simple logistic regression with death and a single component analysed. Models 1 and 2 used death and components in multiple logistic regression. The analyses include all subjects with admissions before the study period and those with multiple admissions

Further analysis is shown in Table 3. It adjusts for prior admission, multiple admissions and age. It demonstrates a consistent interaction of diabetes and age across the adjustments where age is not restricted. However, in Model 6 (see Table 3), where age is limited to 80 years or more, diabetes is no longer a significant component of the regression.

**Table 3:** Logistic Regression for Mortality

	Model 2 (*n* = 139,130)			Model 3 (*n* = 69,617)			Model 4 (*n* = 69,400)			Model 5 (*n* = 42,020)			Model 6 (*n* = 17,461)		
	OR	SE	*p*	OR	SE	*p*	OR	SE	*p*	OR	SE	*p*	OR	SE	*p*
Diabetes	3.15	0.72	< 0.001	3.36	1.04	< 0.001	3.39	1.06	< 0.001	4.64	1.76	< 0.001	4.65	5.4	0.188
Age	1.04	0.00	< 0.001	1.03	0.00	< 0.001	1.04	0.00	< 0.001	1.04	0.00	< 0.001	1.04	0.00	< 0.001
Diabetes x age	0.99	0.00	< 0.001	0.99	0.00	< 0.001	0.99	0.00	< 0.001	0.98	0.00	< 0.001	0.98	0.01	0.182
Female	1.18	0.02	< 0.001	1.13	0.03	< 0.001	1.15	0.03	< 0.001	1.12	0.04	< 0.001	1.10	0.05	0.044
Prior Separations(n)	0.94	0.00	< 0.001				0.91	0.01	< 0.001						
Admission Number	0.93	0.01	< 0.001	0.87	0.01	< 0.001									
CCI 1	0.71	0.02	< 0.001	0.78	0.03	< 0.001	0.72	0.02	< 0.001	0.82	0.03	< 0.001	0.64	0.04	< 0.001
CCI 2+	0.91	0.02	< 0.001	0.97	0.03	0.333	0.89	0.03	< 0.001	0.96	0.04	0.240	0.82	0.05	< 0.001
HADx	1.45	0.04	< 0.001	1.66	0.06	< 0.001	1.65	0.06	< 0.001	1.84	0.08	< 0.001	1.67	0.10	< 0.001
Constant	0.01	0.00	< 0.001	0.01	0.00	< 0.001	0.01	0.00	< 0.001	0.01	0.00	< 0.001	0.01	0.00	< 0.001

Abbreviations: *OR* Odds ratio; *se* Standard error; *CCI* Charlson Comorbidity Index; *HADx* Hospital-acquired diagnosis. Models comprise components shown. Model 2 contains all variables without restriction and is the same as Model 2 in Table 2; Model 3 is restricted to those with no prior admission; Model 4 is restricted to the first admission only; Model 5 is limited to those with no prior admission & first admission only; Model 6 is Model 5 limited to those people older than 80 years (the median age of the cohort)

### Secondary outcomes

People with diabetes had a significantly longer LOS (10.2 days SD (13.4) v 9.4 days SD (12.3), *p* < 0.001 Mann-Whitney). As with mortality, there is an interaction with age with the predicted LOS being lower in people with diabetes beyond the age of 95 years, see Fig. 1b.

There was a small increase in the odds of developing a hospital-acquired complication in people with diabetes (OR: 1.09 SE (0.02), *p* < 0.001). There was no interaction with age or other confounders. The increased odds persisted throughout the age spectrum.

People with diabetes had a 14.0% 28-day readmission rate compared to 12.1% in non-diabetics (*p* < 0.001). Diabetes did not affect 28-day re-admission rate in logistic regression when adjusted for age, sex, prior admissions, other comorbidities and HADx (OR: 0.88 SE (0.18), *p* = 0.523).

### Discussion

A commonly held view amongst experienced geriatricians is that strict adherence to onerous diabetic diets with maintenance of tight blood sugar ranges does not confer morbidity or mortality benefits to very old adults, and comes at a cost to patient quality of life. Clinical decisions to relax glycaemic control in the context of significant comorbidity occasionally leads to conflict with other clinicians or patients and their families. To date, there had been no large studies to help inform such management decisions in very old adults. The present study did not find that diabetes was associated with increased odds of mortality in people aged over 65 years with an unadjusted odds ratio of 0.89 (*p* < 0.001). Diabetes diagnosis has an interaction with age. Accumulating age reduces its impact to the point that a diagnosis of diabetes may predict a better outcome in the very old adults (aged more than 80 years), see Fig. 1a. This may be due to immortal time bias (only the healthiest diabetics survive to ages beyond 80 years) [17, 18]. At the very least, it confirms that diabetes in very old adults does not convey a worse prognosis.

The transition point to the very old adult is arbitrary. By using the median age of the cohort (80 years) as this study’s transition point, additional analysis, restricted to the oldest, showed no effect of diabetes diagnosis on mortality (see Table 3). Most studies that deal with mortality and diabetes are longitudinal studies [19–23]. There are only a few studies that explore in-hospital mortality associated with diabetes, and these are disease-specific [24–26]. Two of the studies showed no effect on inpatient mortality [24, 26]. The third showed an odds ratio of 1.31 (1.04–1.65) for mortality in people with diabetes with foot disease [25]. There may be disease-specific subsets that are at higher risk, but this was not explored in the present study.

Similar to mortality, diabetes diagnosis had less impact on the length of stay (LOS). Greater LOS may be used as an indicator of greater physical and/or psychological morbidity. Very old adults with diabetes had shorter lengths of stay (see Fig. 1b) despite people with diabetes having overall longer LOS. The impact of diabetes on LOS is consistent with other studies [24, 26–28]. The present study differs from these works by including only people over 65 years and adjusting for the confounding of disease burden by using the CCI. Furthermore, also consistent with these studies is the magnitude of the effect on LOS, which is small.

Hospital-acquired adverse events were higher in the diabetic cohort. It was not modified when adjusting for age. This study used a validated but not extensively used method of detection of hospital-acquired diagnosis [16, 29–33]. The present study also found a much higher proportion of HADX overall, probably related to the examination of an older cohort. By contrast, Cromarty et al. were able to show that 29.3% of people with diabetes developed hospital-acquired events compared to 13% of non-diabetics [29]. .

This study demonstrated that the likelihood of re-admission within 28 days of discharge was associated with the diagnosis of diabetes, and these odds were not influenced with increasing age. Caughey et al. identified older people (aged over 85 years) with comorbidities as those most likely to be re-admitted within 30 days [34]. The present study adjusted for the presence of comorbidity and found little difference, providing some support for their result. Dungan identified those with poor glycaemic control as those most likely to be re-admitted [35]. Clinical measures were not undertaken in the present work.

The present study does not show why the diagnosis of diabetes does not appear to have an impact on mortality in very old adults. However, it does support clinical decisions to relax glycaemic control goals in this age group, as has been more broadly recommended in international guidelines [36]. There is evidence that rigid control may not have the benefits seen in younger people [37]. A converse viewpoint is that diabetes may not be as harmful in older people, and so its control does not need to be as tight. These considerations will enhance the adoption of a more measured approach to diabetic care in very old adults, particularly those with comorbidities, with less strict blood sugar ranges and a more liberal diabetic diet. These measures may in turn facilitate maintenance of independence and improved quality of life in this age group.

The current study has limitations. It is a retrospective audit of hospital administrative data. Hospital databases have not been designed for clinical investigations. However, the large amounts of data might be used for association. Several validation studies of hospital discharge data exploring diabetes diagnosis (as used in the present study) have been conducted with a diabetes diagnosis giving positive predictive values from 59 to 93% and negative predictive values from 91 to 99% [38]. These studies suggest that not having a label of diabetes is more accurate. Improved accuracy of the diagnosis of diabetes can occur by linking hospital datasets with other sets such as prescription databases [39]. Linkage studies were beyond the scope of this study but would merit further research. Moreover, a validation sub-study may be useful. Studies also warn about changes in coding rules occurring over time, such as the changes that occurred in the definitions of diabetes in 2011 [40]. This change has resulted in a decreased level of reporting. This study commenced in 2012 for that reason.

This study chose to analyse each admission as a separate event for its primary outcomes. Thus, individuals may be represented multiple times. However, including or excluding those with multiple admissions could be debated. This study analysed with and without multiple admissions, and with and without prior admission (see Table 3). There was no material difference across the different models.

This study did not use specific clinical measures such as medication usage, glycaemic control and measures of frailty. Several small works have examined hospital outcome based on these clinical measures [41–44]. Prospective studies that explore the effects of glycaemic control and frailty on hospital outcomes are needed.

## Conclusion

Consensus amongst clinicians regarding whether to recommend adherence to strict diabetic management regimens in very old patients in hospital is presently lacking. This uncertainty is influenced by concerns of potential impacts on quality of life as well as lack of perceived benefit of potentially onerous management plans. The present work demonstrates that diabetes does not have a negative impact on mortality, LOS or hospital acquired adverse events in the hospitalised very old adults based on data derived from hospital administrative records. This supports clinician decisions to consider less stringent diabetes management plans for their very old patients.

## Data Availability

The datasets used and/or analysed during the current study are available from the corresponding author on reasonable request.

## Abbreviations

ICD-10: International classification of disease 10th revision
ICD-10-AM: International classification of disease 10th revision Australian modification
SE: Standard error
SD: Standard deviation
p: *P*-value
US: United States
USA: United States of America
UK: United Kingdom
NSW: New South Wales
iPM: Patient information manager
DRG: Diagnosis related code
CCI: Charlson comorbidity index
HADx: Hospital acquired diagnosis
SESLHD: South East Sydney local health district
IQR: Interquartile range
LOS: Length of stay
